# In Silico Prediction of *Plasmodium falciparum* Cytoadherence Inhibitors That Disrupt Interaction between gC1qR-DBLβ12 Complex

**DOI:** 10.3390/ph15060691

**Published:** 2022-05-31

**Authors:** Abdul Hafiz, Rowaida Bakri, Mohammad Alsaad, Obadah M. Fetni, Lojain I. Alsubaihi, Hina Shamshad

**Affiliations:** 1 College of Medicine, Umm AL Qura University, Makkah 21955, Saudi Arabia; rabakri@uqu.edu.sa (R.B.); masaad@uqu.edu.sa (M.A.); s44287140@st.uqu.edu.sa (O.M.F.); s44286729@st.uqu.edu.sa (L.I.A.); 2 King Faisal Hospital, Makkah 24236, Saudi Arabia; 3 Prince Sultan Armed Forces Hospital, Madinah 42375, Saudi Arabia

**Keywords:** malaria, *Plasmodium falciparum*, adjunctive therapy, anti-malarial drug, timbal, docking, cytoadherence

## Abstract

Malaria causes about half a million deaths per year, mainly in children below 5 years of age. Cytoadherence of *Plasmodium falciparum* infected erythrocytes in brain and placenta has been linked to severe malaria and malarial related deaths. Cytoadherence is mediated by binding of human receptor gC1qR to the DBLβ12 domain of a *P. falciparum* erythrocyte membrane protein family 1 (PfEMP1) protein. In the present work, molecular dynamic simulation was extensively studied for the gC1qR-DBLβ12 complex. The stabilized protein complex was used to study the protein–protein interface interactions and mapping of interactive amino acid residues as hotspot were performed. Prediction of inhibitors were performed by using virtual protein–protein inhibitor database Timbal screening of about 15,000 compounds. In silico mutagenesis studies, binding profile and protein ligand interaction fingerprinting were used to strengthen the screening of the potential inhibitors of gC1qR-DBLβ12 interface. Six compounds were selected and were further subjected to the MAIP analysis and ADMET studies. From these six compounds, the compounds **3**, **5,** and **6** were found to outperform on all screening criteria from the rest selected compounds. These compounds may provide novel drugs to treat and manage severe falciparum malaria. Additionally. the identified hotspots can be used in future for designing novel interventions for disruption of interface interactions, such as through peptides or vaccines. Futher in vitro and in vivo studies are required for the confirmation of these compounds as potential inhibitors of gC1qR-DBLβ12 interaction.

## 1. Introduction

Malaria caused an estimated 241 million cases and 627,000 deaths in 2020 [1]. Human malaria infections are often non-life threatening and self-limiting. Children who are below 5 years of age, as well as the pregnant women, are the most vulnerable group to severe malaria and deaths. Despite treatment with anti-malarial drugs, the case fatality rate (CFR) in severe malaria is high for both children and adults. Cerebral malaria, the deadliest complication of severe malaria, has been reported to have CFR of 18% in children and 30% in adults [2].

Immunity to malaria is acquired slowly after repeated infections, therefore children living in the endemic areas develop immunity against severe malaria with age. Adults living in the endemic areas may be immune to the severe malaria disease due to the acquired immunity upon repeated infections. Migration to non-endemic countries or decline in local malaria transmission has been implicated in loss of acquired immunity against malaria [3,4]. As the global malaria control efforts are expected to decrease malaria transmission, it is likely that the proportion of severe malaria cases may increase dramatically due to decrease in the acquired immunity against malaria. Moreover, drug resistance poses a serious challenge in malaria treatment and malaria parasite has developed resistance to almost all the drug in use [5]. The issue of drug resistance in malaria is so serious that the World Health Organization (WHO) now recommends combination therapy for malaria treatment to delay the development of drug resistance [6]. Until now, chemotherapy has been the most effective treatment for malaria; however, toxicity and parasitic resistance have prompted scientists to look for new therapies, targets, and inhibitors [7]. The spreading drug resistance in malaria parasites is a major concern for global malaria control efforts. Therefore, there is an urgent need to find novel drugs that can be used to treat malaria and/or reduce the deaths in severe malaria cases [2].

Malaria parasite *P. falciparum* causes the most severe form of malaria and is responsible for majority of the malaria related deaths. The *P. falciparum* infected erythrocytes exhibit unique cytoadherence properties by which the erythrocytes that are infected with the mature stages of *P. falciparum* bind to the host cell and sequester in the host blood vasculature. Sequestration of *P. falciparum* IEs allows these mature stages of the parasite to avoid splenic clearance. Cytoadherence of *P. falciparum* is thought to play an important role in severe malaria pathogenesis. Cytoadherence in the brain and placenta has been linked to severe malaria [7,8,9].

Cytoadherence is mediated by specific protein–protein interactions. A 32 kD human protein gC1qR has been identified as a receptor for cytoadherence of *P. falciparum* to human brain microvascular endothelial cells and platelets [10]. Subsequent studies have implicated cytoadherence to gC1qR with severe malaria pathogenesis [10,11]. The malaria protein that binds with gC1qR has been identified as a DBLβ12 domain of a *P. falciparum* Erythrocyte membrane protein 1 (PfEMP1) [12]. We have reported that gC1qR trimer asymmetrically binds with DBLβ12 through interaction of 21 amino acid residues of DBLβ12 with 24 amino acid residues of gC1qR trimer [13].

Protein–protein interactions (PPIs) play an important role in biological processes including in the pathogenesis of several disease and provides valuable targets for novel anti-malaria interventions. PPI modulators have already been approved and marketed in recent years and some have entered clinical trials [14]. Computational screening provides a valuable tool in drug discovery. The discovery of Venclexta as B-cell lymphoma 2 inhibitor in 2016 by fragment-based screen has shown the importance of PPI targeting [15]. Computational Drug designing techniques in the form of Docking, Virtual Screening, and Simulation have assisted and played a significant role in drug discovery for hit identification and lead optimization. Computational techniques are less costly and are more direct with effective screening [16]. Furthermore, it has been suggested that PPI inhibition by peptides, peptidomimetics, or small-molecule inhibitors may have the potential to interfere with binding domains for malarial targets [14]. The PPIs involved in host pathogen interactions are considered important targets for novel anti-malarial interventions [17].

Here, we have used a molecular docking study to identify potential drug molecules that may block the interaction between gC1qR and DBLβ12. We have used PPI inhibitor-focused Timbal molecular library [18] for the docking studies and identified potential drug molecules that may be used to block the interaction between gC1qR and DBLβ12. These molecules may act as lead novel drug molecules to treat or manage severe malaria. This is the first study that used DBLβ12 in complex with gC1qR to exploit the drug discovery process in malaria. The docked inhibitors could be used in future study to test and validate as novel interventions against *P. falciparum* malaria.

## 2. Results

### 2.1. Molecular Dynamics (MD) Simulation Analysis of DBLβ12 and gC1qR: Pre- and Post-Complexation State

Molecular Dynamics (MD) simulation analysis is used as a tool to measure the stability, fluctuation, compactness, and conformational alterations of our proteins in apo and complexed states. MD simulation was done for the apo state of DBLβ12 and complexed state when DBLβ12 bound with gC1qR. These two Systems were used for assessment of apo and complexed state. The 100 ns MD production run of each system accomplished for the above-mentioned analytic parameters. 

**Figure 1:**
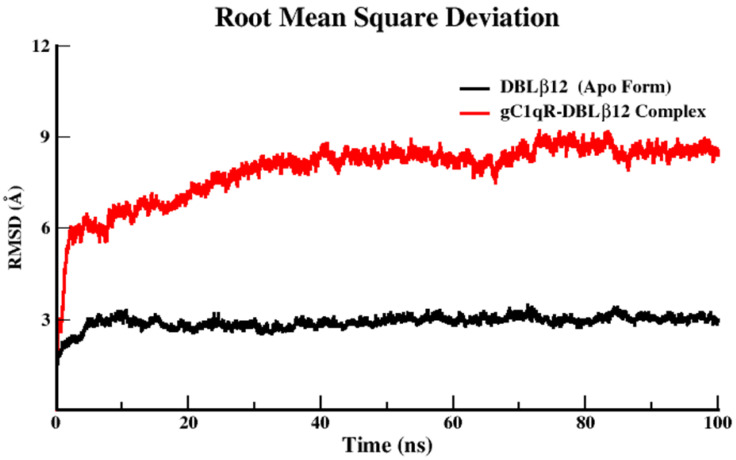
RMSD Analysis of the simulated trajectories upon 100 ns time.

**Figure 2:**
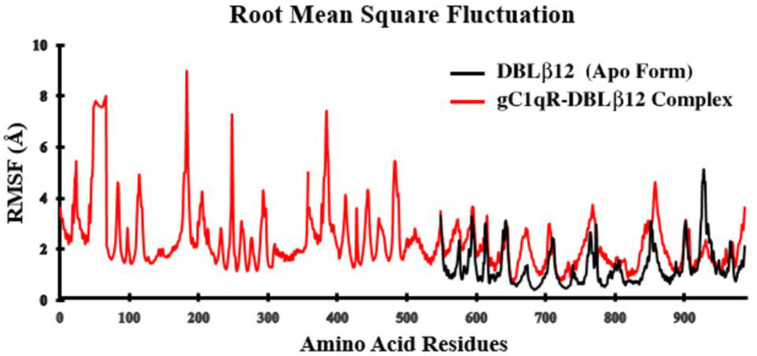
RMSF Analysis of the simulated trajectories upon 100 ns time.

The root-mean-square deviation (RMSD) analysis of the backbone atoms of the uncomplexed DBLβ12 demonstrated a slight deviation with the value of 2.7–3 Å at 100 ns. While after complexation of DBLβ12 model with gC1qR, we observed a drastic deviation with increase RMSD value in the range of 6–8 Å. The relative deviation analysis elaborated that after 20 ns time of MD simulation, the DBLβ12 model showed noticeable stability with the decrease RMSD value of 0.3 Å. Figure 1 illustrates that the deviation in the apo state was quite stable and after complexation the deviating score inflated with the drastic alterations. Another MD simulation parameter root mean square fluctuation (RMSF) estimates the average fluctuation of the protein residues throughout all-atom based simulation. The RMSF was executed on the backbone Carbon atoms in both apo and complexation state. The RMSF analysis was depicted in Figure 2. The RMSF plot revealed that in the apo state, when the DBLβ12 domain didn’t bind to the human gC1qR, the conformation fluctuation was noticeably normal with the range of ±3–4 Å. While in the complexed state, the deviation peak was noted in the B chain of gC1qR trimer. It was evident from MD simulation analysis that the conformational and configurational changes occur when the DBLβ12 domain attract towards gC1qR, and the fluctuations tend to increase in the complexed state of the protein–protein interaction. In the RMSF plot, the amino acid residue starting from 1–544 corresponded to human gC1qR while 545–985 residues indicated the DBLβ12 domain. On the basis of fluctuations, it can be stated that gC1qR Chain B was strongly associated in the interface region as compared to Chain A and Chain C. The degree of fluctuation was noticeable at 545–630 residue during whole simulated trajectory analysis. 

**Figure 3:**
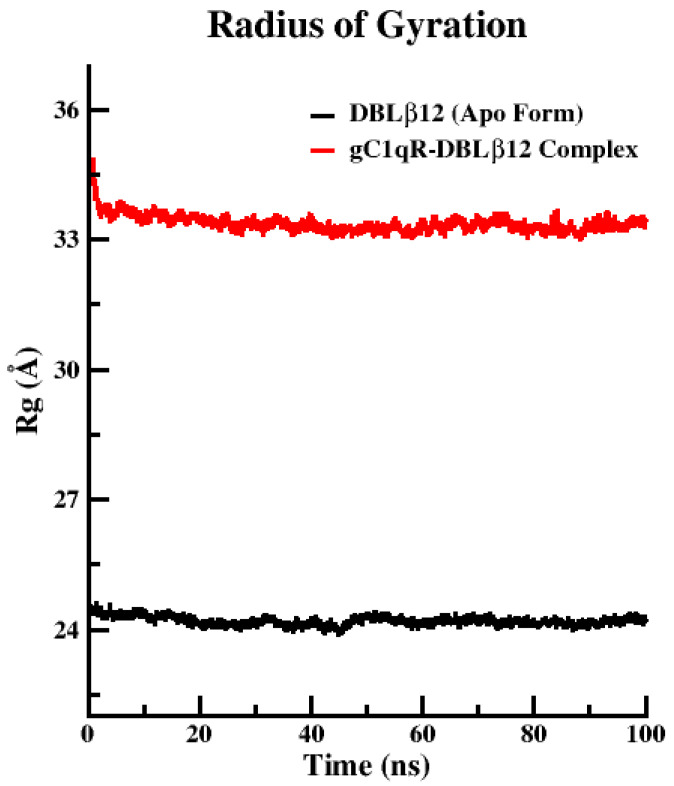
Radius of Gyration Analysis of the simulated trajectories upon 100 ns time.

The folding pattern and compactness of the protein is inferred from the radius of gyration (Rg) calculations in both the apo and complexed states. Folded protein depicts the tight packing and unfolding of protein demonstrates loose packing with less stable conformation and have higher radius of gyration values. Rg plots, as described in Figure 3, clearly demonstrate that apo state of DBLβ12 domain showed more compactness as compared to the complexed state. The Rg for Apo state was estimated to be between 24.3 and 25.5 Å. On the other hand, the complexed state Rg score lay in the interval difference between 34.7 to 34.5 Å. 

### 2.2. Alanine Scanning Mutagenesis (ASM) Analyses of DBLβ12 and gC1qR

**Figure 4:**
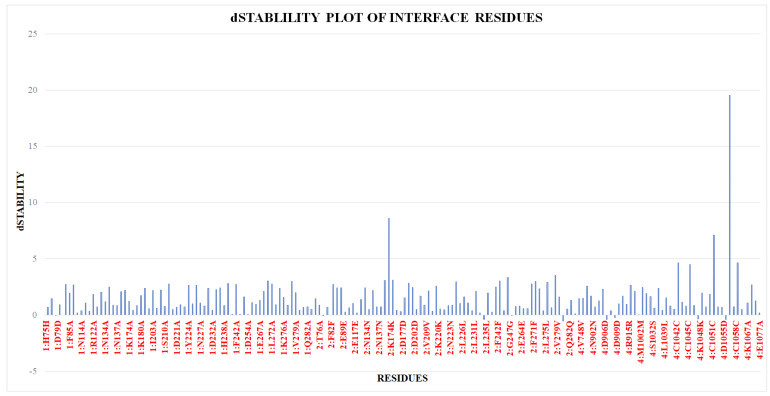
dStability values of Interface residues of the complex, where 1: Chain A of DBLβ12, 2: Chain B of DBLβ12, 4: gC1qR.

Alanine scanning mutagenesis is an in silico approach to estimate the role of specific residue in the stability of the protein–protein complex and the functional importance of these residues [19]. This technique calculates the relative affinity and stability of the mutant and correlate the wild type. The negative value of the stability indicates the more stability of the complex. Hence, a positive value of the dStability shows the importance of the particular residue in the interface region of the complex structure [20,21]. Alanine Mutagenesis studies was conducted using MOE analyse the role of specific amino acid in the stability of the DBLβ12 and gC1qR [19]. The crucial interface residues involved in the interface regions were identified for the gC1qR and DBLβ12 using dStability values as depicted in the Figure 4.

Post-identification of the crucial hotspot interface residues using MOE [22], cross-validation were further assessed by PPCheck [23] and DrugScore^PPI^ [24] webservers were further utilized to validate and select the important hotspot residues. The results of the webservers and Alanine scanning via MOE [22] were correlated and the selection of the important residue were noted for the subsequent docking studies. DBLβ12 residues L747, M1002, K1048, Q1049, and I152 and gC1qR residues F85, H238, and D254 were found to be common hotspot residues in all the analysis. To find a patch for ligand binding with hotspot residues patch maker embedded in MOE was utilized. It was observed that K1048, Q1049, and E1176 were found to make one patch hence these residues were chosen for the interface binding site of the ligand. Patch maker creates surface properties as hydrophobic and electrostatic potentials and helps in the identification of potential reactive sites for oxidation and deamidation. Three residues K1048, Q1049, and E1176 of DBLβ12 were selected as they were found to be located in close proximity and were also very close to the chain B residues D254 and E258. The residues between 188 and 259 have also been reported to be more frequently attached with the other proteins (Hepatitis C virus) [13]. Hence, this site was selected to look for the novel ligand so that maximum interacting hotspot residues can be utilized. 

### 2.3. Docking of the Virtual Database

Identified crucial hotspots residues K1048, Q1049, and E1176 of the DBLβ12 and gC1qR residues D254 and E258 were treated as binding region to dock the database of ~15,500 compounds using MOE against DBLβ12-gC1qR complex. The Timbal database comprised of compounds that have previously shown binding and inhibitory activities with the various protein–protein disrupting functions. Post docking analysis was performed using protein ligand interaction fingerprints (PLIF) tool, to enhance the outcome of docking performances. For fingerprinting, two types of interactions can be categorized: one is based on potential energy, while the other is surface contact based between residue and molecule. Both methods were used to prioritize the virtually scanned hits. A fingerprint clustering technique was used to prioritize the hits and identify the inhibitors. Two step filters were applied to narrow down the virtual data to conclude with the highest yielding leads. In the first step, the score-based filtration was executed. Subsequently, in the second step, PLIF based filtration was used with the specified hotspots residues. The scored based filtered with minimum of one hotspot residues yielded 10,057 compounds. From these 10,057 compounds, the top 3 compounds were selected on the basis of binding affinity score. Top-ranked score based assorted data were further assessed with hotspot residue which was taken into an account to taper off the virtual data. PLIF analyses with the specific hotspot resulted with the 361 number of compounds. Elucidation of the top-ranked potential leads, the examination of the PLIF mining data were investigated by deep molecular interaction analysis. 

Hence forth, S score, fingerprinting, and visual interactions, especially with the hotspot residues, were used to select the top-ranked cherry hits. The topmost six docked compounds with the properties as shown in Table 1 were further subjected to resistance scan (mutagenesis studies)–implemented in MOE. Low mode MD was used to generate ensemble for designing all possible mutations of the selected residue using Resistance Scanning of MOE. 

### 2.4. Molecular Investigation of the Top-Hits to Disrupt Protein–Protein Interactions

**Figure 5:**
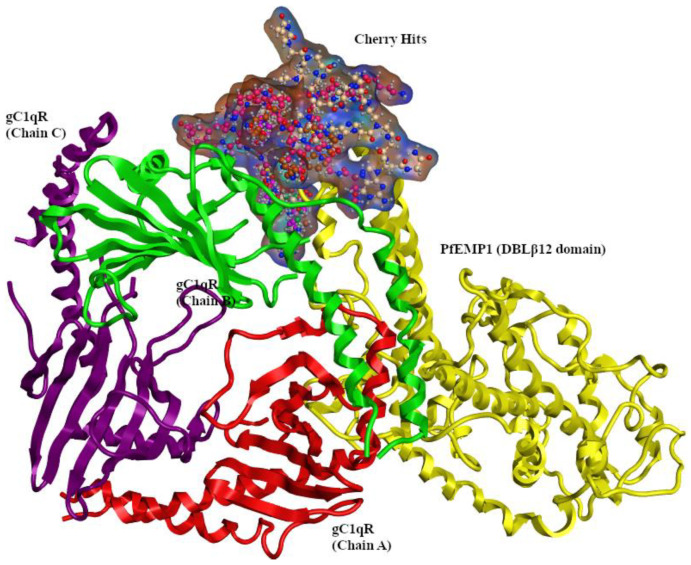
Overlay representation of the cherry hits disrupting DBLβ12-gC1qR.

Molecular investigation of the cherry hits was carried out to observe the possible interaction occurred between both proteins via disruptors. The cherry hits reside in the same binding region and the probable ligand binding interactions was observe with the gC1qR (B chain) and DBLβ12. The overlay representation of the cherry hits also illustrated in Figure 5. Compound **1** (ChEMBL1241020) established hydrophobic interactions with residues as K1050, T1052, and V1056 while hydrogen bonding was observed with H99, K100, T101, I203, N250, T251, Q1043, Q1049, T1052, and D1055. In the case of compound **2** (ChEMBL1269294), hydrophobic interactions were observed with Q1049 and E1179, while hydrogen bonding was observed for K100, T101, K104, T163, D202, K1048, Q1049, K1050, and C1183. Compound **3** (ChEMBL1938611) involved I96, K98, H99, T163, K1050, T1052, E1053, D1055, D1057, and C1183 residues in hydrogen bonding. Compound **4** (ChEMBL58763) was found to make hydrophobic interactions with T163, T165, Y188, and E1053 while hydrogen bonding was observed for T165, D202, E1053, E1176, and C1183. D254. Hydrophobic interactions in the case of compound **5** (ChEMBL310965) were observed for T163, T165, I203, K1048, T1052, E1176, E1179, K1048, and 1075, while D254 was found to make hydrogen bonding. In Compound **6** (ChEMBL327274), T163, T165, T251, K1050, E1176, and E1179 made hydrophobic contacts, and D254 was involved in hydrogen bonding. 

**Figure 6:**
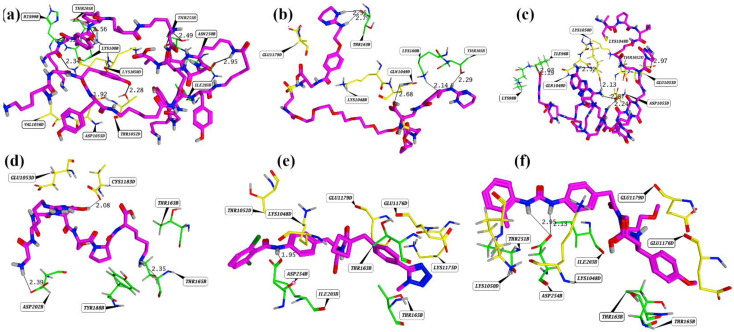
Molecular representation of the top-ranked PPI inhibitors against DBLβ12-gC1qR. (**a**–**f**) represents compound **1** to **6**, respectively.

From the above interactions, it was inferred that H99, K100, T101, T163, T165, T251, and D254 were found to form most of the interaction with the compounds. T163, I202, and D254 were mostly involved in hydrogen bonding contacts with most of the compounds. K1050, T1052, E1053, and D1055 were mostly involved in the hydrogen bonding, while K1048 mostly was involved in the hydrophobic binding. Compound 3 was found to make maximum hydrogen bonding with the interface residues of DBL12-gC1qR complex. Apart from hotspot residues, the interaction with reported unique interface residue T163 was also observed with all the selected compounds. Moreover, binding of the compounds with few of the 188–259 residues were also observed, which have been reported to be involved in the most frequent binding of gC1qR with other proteins, such as hepatitis C virus core protein [13] (Figure 6). The IUPAC naming and structures of the selected compounds are shown in Table S1 [25].

### 2.5. Alanine and Resistance Scan of the Top-Ranked Hits

**Figure 7:**
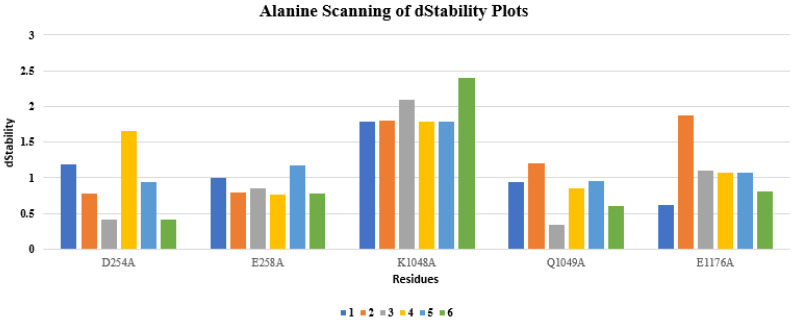
Alanine scan applied on the cherry hits using dStability score.

**Figure 8:**
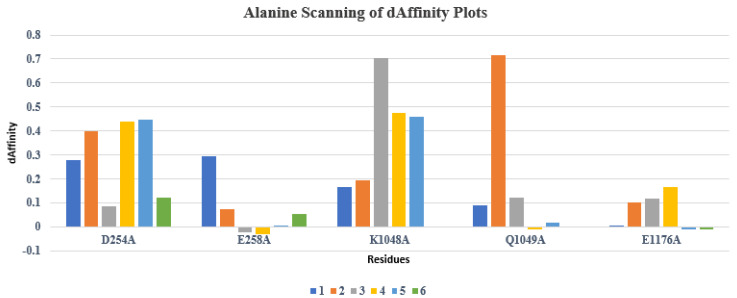
Alanine scan applied on the cherry hits using dAffinity score.

Alanine scanning using GBVI/WSA dG scoring function implemented in MOE was also performed on the top cherry hits bound DBLβ12-gC1qR complex one by one on the selected five residues. The resultant changes in the ligand binding affinities and complex stability were evaluated as shown in the Figure 7 and Figure 8. Among all the compounds, the **4**, **5**, and **6** compounds were found to perform better than compounds **1**, **2** and **3**. The affinity values of the **4**, **5**, and **6** compounds were found to be less positive than other compounds. Moreover, compound **6** was found to be best among all the selected compounds as it indicated more negative affinity values for the selected residues. 

**Figure 9:**
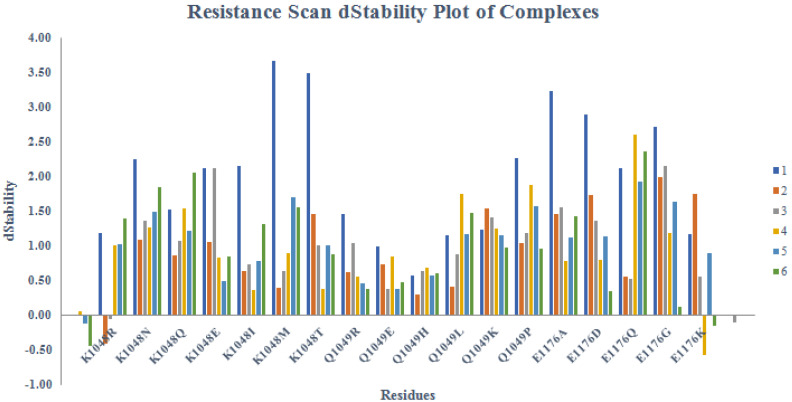
Resistance scan dStability profile of the identified hotspot residues in cherry-picked hits.

**Figure 10:**
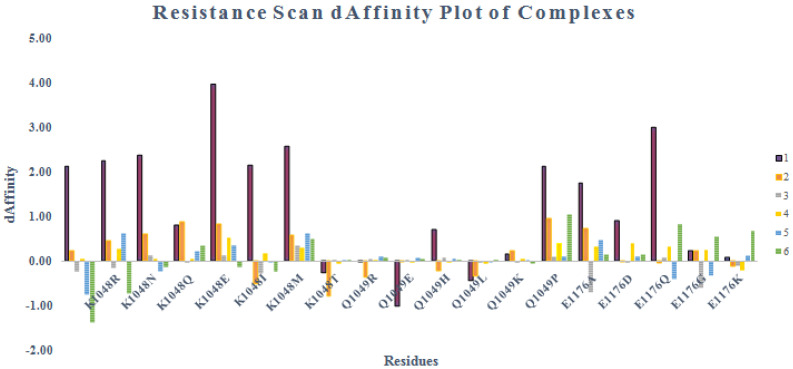
Resistance scan dAffinity profile of the identified hotspot residues in cherry-picked hits. (Colored box 1–6 represent compound **1** to compound **6**, respectively).

Resistance scan profile on the basis of dStability and dAffinity score demonstrated in Figure 9 and Figure 10. The resistance scan was conducted on the docked cherry hits selecting DBLβ12 three hotspot residues, and binding affinity were calculated in contrast to the wild type and mutant residues. All mutations were carried out sequentially and their affinities were calculated. Affinities with large positive increase indicated that the target becoming resistant to the ligand upon mutation with the respective SNP at that position.

The results of ensemble-based resistance scan were found to be similar as the case with Alanine scanning as PLIF based compounds were found to be better than others. However, compound **3** was found to outperform all the other compounds in affinities for resistance scan. It was 9/8 observed that K1048 mutations resulted in positive increase affinity values of all the compounds. However, for compounds **3** and **6**, the values were found to be more negative and slightly positive. The mutations of Q1049 resulted in better affinity profile for all the selected compounds. While E1176 mutations conferred positive increase in the affinity values of all the compounds except for the compound **3**. Overall, compounds **3**, **5** and **6** were found to give the best results, i.e., affinity in these compounds was found to be less positive compared to other compounds. Hence, based on resistance scan, compounds **3**, **5** and **6** compounds can confer less resistant to the target upon mutations. Hence, from Alanine scanning and Resistant scanning, compounds **3**, **5** and **6** can be further tested for their activity against the target. However, stability upon mutation for the compounds **3**, **5**, and **6** were found to be low as compared to other compounds, while stability upon mutation was highest in case of compound **1** as shown in Figure 3. 

### 2.6. ADMET Profiling of Compounds

Absorption, distribution, metabolism, excretion, and toxicity (ADMET) defines the pharmacokinetic and toxicity profile of compounds and is important to deduce the effectiveness and pharmacological properties of the new therapeutic agent against a target as well as its safety profile [26].

The absorption parameters in case of water solubility showed no specific difference among the compounds. However, intestinal absorption was shown only by compounds **5** and **6**. None of the compounds were found to inhibit p-glycoprotein I or II. Subsequently, compound **5** was found to be inhibitor of p-glycoprotein II. Moreover, except for compound **5**, all the other compounds were found to become substrate for p-glycoprotein. BBB and CNS penetration was found to be present with all the compounds, thereby indicating that all the compounds can effectively work against cerebral malaria. It was observed that only compounds **5** and **6** can have the ability to become Cytochrome P450 3A4 (CYP3A4) substrates and both of these compounds can also become inhibitors of CYP2C9. The total clearance was found to be highest for compound **1** and lowest for compound **5**. The toxicity profile of AMES test showed only compound **3** to be active as mutagen while for the rest of the compounds it was negative. 

Compounds **1**, **3**, and **5** (Table 1) were found to be inhibitors of hERG II while for the rest, it was negative. LD_50_ for oral rat acute toxicity was null for all compounds; however, Oral Rat Chronic Toxicity was found to be highest for compound **1**. Hepatotoxicity was absent in compounds **3** and **4**, while others were found to show hepatotoxicity. The results of ADMET for all the compounds are shown in Table S2. 

### 2.7. MAIP Analysis

**Figure 11:**
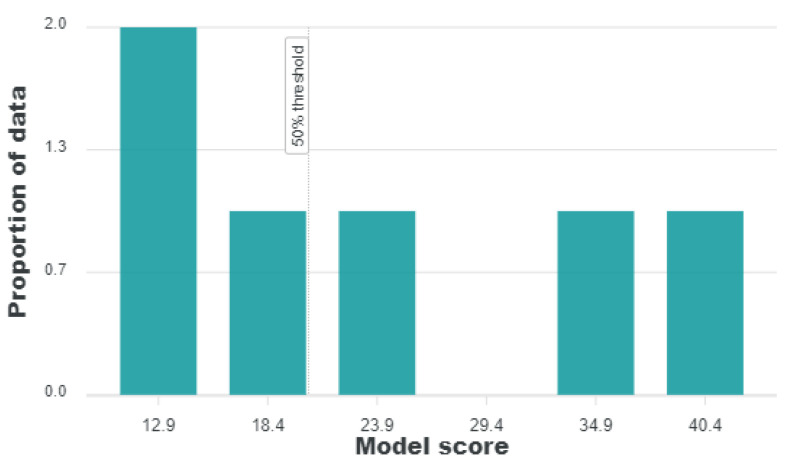
MAIP analysis of the top-ranked cherry compounds enriched in the Anti-malarial dataset implemented in ChEMBL database.

**Figure 12:**
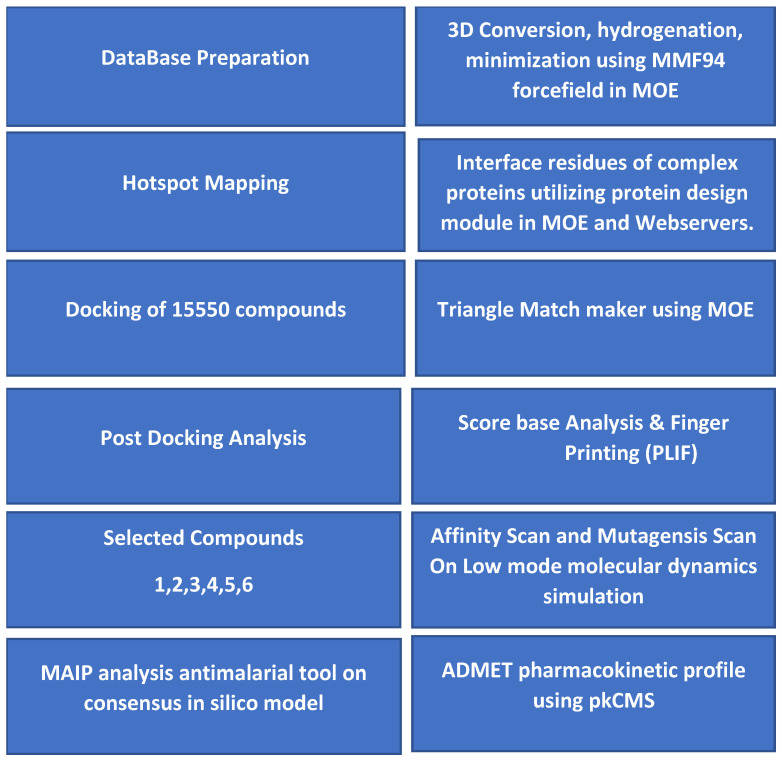
Schematic representation of the virtual screening protocol.

ChEMBL Database was used to predict the Antimalarial inhibition profile of the selected compounds on three different datasets as MMV, PubChem, and St. Jude sets [27]. The Enrichment factor is based on early detection of actives within the list of compounds [28]. All the six compounds yielded modest results Table 2 and Figure 11. The scores for enrichment factors are shown between parentheses. However, the higher the score the higher will be enrichment and it depends on the dataset size. Among all the sets MMV test sets are more enriched with antimalarial compounds, thereby having lower enrichment than others while ROC curve (Receiver operating characteristics) values close to 1 indicates good selectivity. Schematic representation for the virtual screening and selection of compounds is shown in Figure 12. 

## 3. Discussion

Malaria is the most important parasitic disease major public health problem in the tropical world. The malaria parasite is a complex pathogen, and it has been proven difficult to develop an effective vaccine or identify novel drugs against malaria. Additionally, the malaria parasite has been reported to develop resistance to several anti-malarial drugs in different parts of the world. The *P. falciparum* Erythrocyte Membrane Protein 1 (PfEMP1) is a family of proteins encoded by *var* genes. About 60 *var* genes are present in a single *P. falciparum* genome encoding different PfEMP1. PfEMP1 proteins are known to mediate cytoadherence. PfEMP1 proteins are also have important role in antigenic variation of *P. falciparum.* Expression of *var* genes is switched with erythrocytic cycles providing antigenic variation and changed cytoadherence properties. Through cytoadherence, PfEMP1 proteins directly support parasite survival through avoidance of splenic clearance. Therefore, targeting PfEMP1 for novel anti-malarial intervention is likely to be an effective strategy for management of severe malaria cases.

However, targeting of PfEMP1 is challenging since there are about 60 different proteins in a single *P. falciparum* genome and their expression is switched. Moreover, not all PfEMP1 mediated cytoadherence phenotypes are associated with severe malaria. Our and others work has established that gC1qR is an important cytoadherence receptor for *P. falciparum* involved in pathogenesis of severe malaria. The PfEMP1 binder for gC1qR mediated cytoadherence has been identified as PFD0020c.

The reason for incorporating molecular dynamics results is to observe how the crucial hotspot residual changes affect the conformational or structural change, its impact in stability profiling. Furthermore, the aim of the MD study is to reveal the association of both proteins with respect to time providing the physiological conditions. Henceforth, it has been predicted by correlating the RMSD and RMSF results that gC1qR tri-monomeric receptor Chain B directly associated and showed discrete changes with respect to 100 ns of the speculated time duration. The Rg value signified that the overall compactness and stability of the associated protein–protein complex of the simulated system was very low. It also clearly demonstrated that when DBLβ12 domain bound to gC1qR increase in Rg score which elaborate the stability was unattained and compactness retained to be loose during the 100 ns of MD production runs. Overall, MD result analyses suggested that we need to design the disruptors, which may interrupt the association DBLβ12 domain and hum. These MD simulation results clearly suggest that when the DBLβ12 domain is bound to the gC1qR, it increases in Rg score which demonstrate that stability was unattained, and compactness remained loose during the 100 ns of MD production runs. The MD result suggests that we need to design the disruptors, which may interrupt the association of the DBLβ12 domain and human gC1qR.

PPI has been identified as an emerging new therapeutic target and has a high prevalence in the human body compared to a single protein. PPI has different physicochemical features compared to single protein targets. Therefore, a number of PPI inhibitors have been approved or are in the stage of clinical trials [29]. In recent years, many PPI inhibitors have been screened against PPI targets and considerable success has been achieved in the treatment of different diseases [30]. 

It has been reported that the protein–protein interface is wide, flat, and contains an array of polar and non-polar contacts over a large area. Small molecules, therefore, cannot bind tightly to such surfaces as, in the absence of concavity, it can only bind to one side of the binding site [31]. However, PPI drugs do not follow Lipinski’s rule and differ from the conventional rules. Hence, it has been suggested to design a new rule for PPI-drug discovery [29]. In the same way, the drug against Bcl-2/Bcl-XL has a molecular weight greater than 1100 Daltons [18]. Hence, the PPI drugs have higher molecular weights compared to conventional drugs. It is believed that in future, more PPI modulators may be developed to aid in different diseases [14].

This study has identified potential inhibitors that may block interaction between cytoadherence receptor gC1qR and its known binder protein DBLβ12 of PfEMP1 PFD0020c. This is the first study that reports potential inhibitors of gC1qR-DBLβ12 interaction in malaria.

The present paper deals not only the structural prediction of protein–protein complex, their interface interaction, but also identifies relevant interactive amino acid residues as hot spots which can be used for designing of the peptide or compounds to disrupt the interaction of complex. Binding of protein–protein complex can be attributed to a small group of amino acids termed as hotpots residue, which were crucial in the attachment of any protein–protein or protein-ligand association. Identification of these interface residue hotspots were very important in design on interaction inhibitors [29,30]. Potential inhibitors have been identified in the present work after scanning of 15,000 PPI library of inhibitors. We used mutagenesis studies to supplement hotspot identification. Among the six compounds that we report as potential inhibitors of protein–protein interaction, the compounds **3**, **5**, and **6** were found to perform better in interaction profiling, mutagenesis, and resistance scan studies. Further in vitro studies are needed to establish their potential to inhibit the gC1qR-DBLβ12 interaction.

## 4. Materials and Methods

### 4.1. Molecular Dynamics Simulation Protocol Applied for the Generation of DBLβ12-gC1qR Complex

MD Simulation protocol was followed to explore the structural traits and dynamic state alterations utilizing docked pose of Duffy binding-like β12 (DBLβ12) domain of *Plasmodium falciparum* erythrocyte membrane protein 1 (PfEMP1) with the associated target Human receptor gC1qR previously reported by Hafiz et al. Apo state of the DBLβ12 and gC1qR MD simulation of 100 ns run were carried out to correlate the conformational dynamics in apo and complexed states. MD simulation software Gromacs 2021.2 (Version) [32] was used in the current study. Pdb2gmx tool in Gromacs was utilized for the topological generation using the amber 99 (Force field) to the provided co-ordinate of DBLβ12-gC1qR Complex PDB file. The complex system was further subjected for the solvation in the aqueous medium using simple point charge (SPC) water model. Additionally, periodic boundary conditions (PBC) were set to abstain the structural surface artifacts during simulation. Neutralization of the system was preceded by adding 25 Na^+^ counter ions using genion module prior to energy minimization step. A total of 50,000 energy minimization steps against DBLβ12-gC1qR complex were further followed to avoid hindrance and steric clashes. Moreover, an equilibration step was processed against DBLβ12-gC1qR Complex retaining 300 K temperature with constant volume and pressure for the timescale of 100ps followed by two steps NVT and NPT ensembles. A Linear Constraint Solver for molecular simulations algorithm implemented in Gromacs was subsequently used for the fixation of the bond lengths with the 10 Å cut-off range. Furthermore, the Particle Mesh Ewald (PME) method was applied for computing long-range electrostatic interaction. After the Equilibration step, 100 ns of MD production run was accomplished to generate snaps shots to monitor the conformational trait and stability of DBLβ12-gC1qR complex. The MD Simulations analysis parameters were used for further inspections. XMGrace Software [33] was further used to elucidate the stability, fluctuation, compactness profiling plots of the complex. Visual inspection of the 100 ns trajectory analyses of apo and complex system were taken into account by using VMD software [34].

### 4.2. Alanine Scanning Mutagenesis (ASM)

In silico alanine scanning mutagenesis studies were executed by multiple softwares to predict the core residues identification which could lead to hot spots or the warm spots into the targeted protein. The purpose of carrying mutagenesis studies is to observe the role of the crucial residues in terms of function and stability profiling. The post-MD coordinate PDB file was further submitted to MOE software and the other web-servers such as DrugScore^PPI^ [24] and PPCheck [23] for the alanine scanning study. These approaches are rapid, accelerable, and reliable, which employ free energy function to anticipate the hot spots based on knowledge-based potentials. It also calculates the mutational impact on a protein by the computing the stability of a complex from wild type to mutant type. For instance, protein–protein interface residues of gC1qR and DBLβ12 in the vicinity of 5 Å zone were utilized for the Alanine Scan using Protein Design module implemented in MOE2019 [22]. For further validation, DrugScore^PPI^ [24] and PPcheck web-servers [23] were also used to identify the hotspot residues and to correlate the results. 

### 4.3. Virtual Database Preparation

Timbal Database [18] is the first hand-curated database dedicated to small molecule protein–protein interaction inhibitors and provides general molecular properties of the modulators. Hence, it was used to search the potential protein–protein inhibitor termed as PPI against DBLβ12-gC1qR. The downloaded database was retrieved from publicly accessible website [18]. Furthermore, the retrieved database was subjected to molecular conversion from 2D to 3D pattern. The generated 3D structural database was further cleaned, hydrogenated, and minimized by using forcefield MMFF94X implemented in the Ligand Prep module presented in MOE.

### 4.4. Molecular Docking and Virtual Screening

Prepared PPIs from Timbal database against DBLβ12-gC1qR complex were selected for subsequent virtual screening using MOE-Dock Program. The triangle matcher was used as a placement method with 05 final docking output. The predicted hotspot residues of the interface region were used to discover the binding site. The prepared chemical library of ~15,550 compounds were used to screen the disrupting effects in the interface region of two proteins using default docking parameters. Score-based analysis was performed to assort the top-listed compounds of the chemical library. Second filtration was applied on the basis of the potential hotspots identified in early stage of the study on the top-binding score data [35]. Fingerprinting criteria was further introduced to sort the data and the PLIF calculations presented in MOE was used to observe the molecular interactions with the identified hotspot residues. Hereafter, these filters were used for the selection of the top-scoring and hot-spot residual binding interaction. Deep Molecular Interaction analysis of the cherry hits was performed on MOE [22] and discovery studio [36].

### 4.5. Affinity and Resistance Scan

The affinity and stability of the top-rank docked compounds were analyzed by generating the protein confirmations ensemble using a low mode molecular dynamic simulation on MOE [22]. Low mode molecular dynamics simulation was performed for 1 ps and a confirmation have been saved for every 5 ps. 

A resistance scan was also carried out using low mode molecular dynamic simulation on the five hotspot residues. In this case Single Nucleotide polymorphisms at each point was used for the crucial hotspots’ residues and the effect on the ligand binding affinity was computed. Thus, the resistance scan was also utilized to specify the mutation of the selected residues and their affinity was calculated. Large positive value indicated that target may become resistant to the ligand upon mutation.

### 4.6. ADMET Profiling

The freely available web server pkCMS was used for the prediction of pharmacokinetic profile of the selected compounds. The methodology of this webserver involves graph-based signatures for the prediction of pharmacokinetic properties [26].

### 4.7. MAIP Analysis

The antimalarial tool MAIP [27] based on consensus in silico model implemented in ChEMBL webserver was used for the large-scale prediction of antimalarial activities of the compounds. The MAIP is based on open-source tools and is freely available for the prediction of compounds which is integrated with the ChEMBL website.

## Figures and Tables

**Table 1:** Properties of the selected compounds.

Compounds Name	ChEMBL Code	LogP	Rotatable Bonds	H-Bond (Acceptor)	H-Bond (Donors)	Binding Score
**Compound 1**	1241020	−1.91	89	34	34	−14.34
**Compound 2**	1269294	−3.2	50	27	12	−13.36
**Compound 3**	1938611	−17.42	68	54	22	−13.24
**Compound 4**	58763	−6.27	23	12	12	−9.88
**Compound 5**	310965	4.96	10	7	3	−9.35
**Compound 6**	327274	4.38	15	6	4	−9.13

**Table 2:** MAIP analysis of selected docked compounds on three different datasets.

Performance Metrics	MMV Test Set	PubChem	St. Jude Screening Set
**ROC AUC score**	0.67	0.69	0.81
**EF [1%]**	3.5 (60)	7.0 (56)	12.1 (71)
**EF [10%]**	2.1 (41)	2.8 (47)	4.8 (36)
**EF [50%]**	1.4 (23)	1.5 (34)	1.8 (15)

## Data Availability

Data is contained within the article and Supplementary Material.

## Supplementary Materials

The following are available online at https://www.mdpi.com/article/10.3390/ph15060691/s1, Table S1: IUPAC names and structures of the compounds. Table S2: ADMET parameters for the selected compounds.
