# Supplementary material for: In Silico Prediction of Plasmodium falciparum Cytoadherence Inhibitors That Disrupt Interaction between gC1qR-DBLβ12 Complex
Source: Pharmaceuticals (Basel). 2022 May 31;15(6):691. doi: 10.3390/ph15060691 (PMC9230678; doi:10.3390/ph15060691)
Supplement: Supplementary file 1 [file pharmaceuticals-15-00691-s001.zip › pharmaceuticals-1677279-supplementary.pdf]

SUPPLEMENTARY FILES

**Table S1.** IUPAC names and structures of the compounds (25)

| Name | IUPAC Name |
|---|---|
| 1 | (2R)-2-[[(2S)-2-[[(2R)-2-[[(2S)-2-[[(2R)-2-[[(2S)-2-amino-6-[6-[[(2S)-2-[[(2S)-2-[[2-[[(2S)-2,6-diaminohexanoyl] amino]acetyl]amino]-3-(4-hydroxyphenyl)propanoyl]amino]-3-(4-hydroxyphenyl) propanoyl] amino]hexanoylamino]hexanoyl]amino]propanoyl]amino]-6-[6-[[(2S)-2-[[(2S)-2-[[2-[[(2S)-2,6-diaminohexanoyl] amino]acetyl]amino]-3-(4-hydroxyphenyl) propanoyl] amino]-3-(4 -hydroxyphenyl) propanoyl] amino] hexanoylamino] hexanoyl] amino] propanoyl]amino]-6-[6-[[(2S)-2-[[(2S)-2-[[2-[[(2S)-2,6-diaminohexanoyl]amino]acetyl]amino]-3-(4-hydroxyphenyl) propanoyl]amino]-3-(4-hydroxyphenyl) propanoyl]amino] hexanoylamino] hexanoyl] amino] propanoic acid 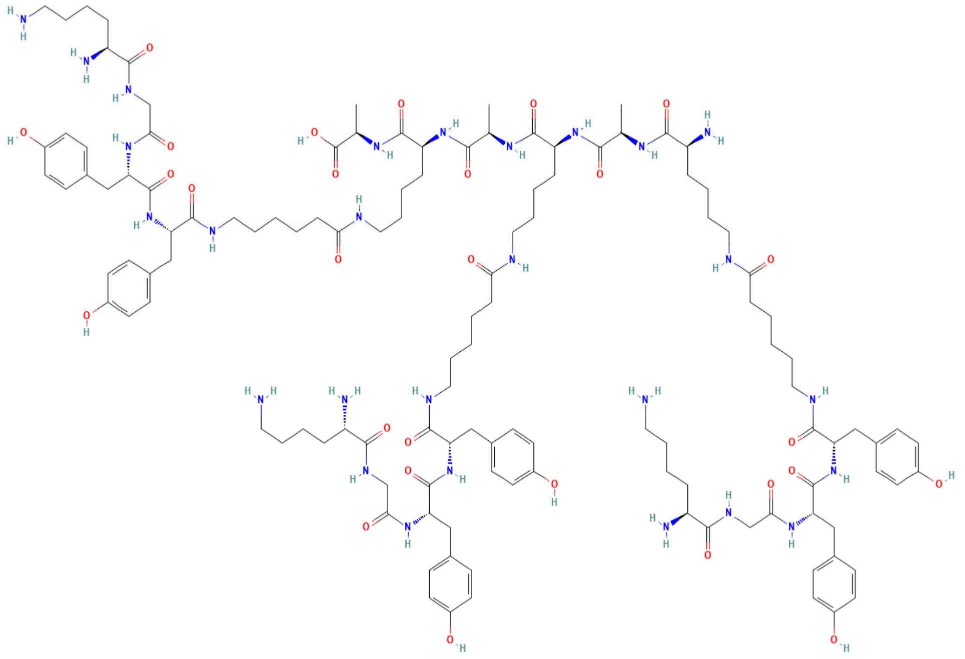 |
| 2 | (2S)-2-[2-[2-[2-[2-[2-[2-[2-[2-[2-[2-[[(1S)-1-carboxy-2-[[4-[2-(1,4,5,6-tetrahydropyrimidin-2-ylamino)ethoxy]benzoyl]amino]ethyl]sulfamoyl]ethylcarbamoyloxy]ethoxy]ethoxy]ethoxy]ethoxy]ethoxy]ethoxy]ethoxy]ethoxycarbonylamino]ethylsulfonylamino]-3-[[4-[2-(1,4,5,6-tetrahydropyrimidin-2-ylamino)ethoxy]benzoyl]amino]propanoic acid 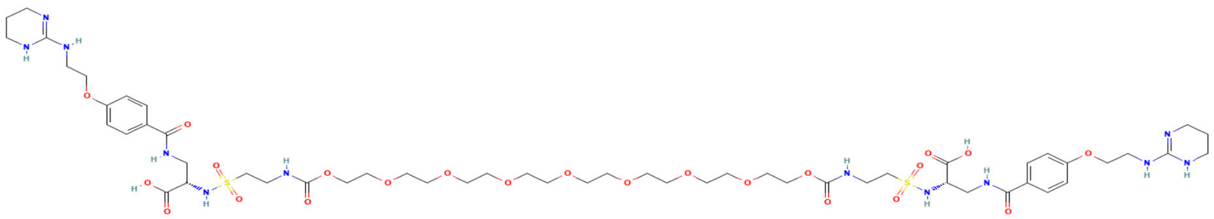 |

SUPPLEMENTARY FILES

3 (2S)-N-[(2S,3S)-1-[2-[2-[2-[2-[[2-[2-[[2-[2-[[2-[2-[[2-[2-[[2-[2-[[2-[2-[[2-[(2-amino-2-oxoethyl)amino]-2-oxoethyl]-[2-(2-amino-6-oxo-1H-purin-9-yl)acetyl]amino]ethylamino]-2-oxoethyl]-[2-(6-aminopurin-9-yl)acetyl]amino]ethylamino]-2-oxoethyl]-[2-(4-amino-2-oxopyrimidin-1-yl)acetyl]amino]ethylamino]-2-oxoethyl]-[2-(6-aminopurin-9-yl)acetyl]amino]ethylamino]-2-oxoethyl]-[2-(5-methyl-2,4-dioxopyrimidin-1-yl)acetyl]amino]ethylamino]-2-oxoethyl]-[2-(4-amino-2-oxopyrimidin-1-yl)acetyl]amino]ethylamino]-2-oxoethyl]-[2-(4-amino-2-oxopyrimidin-1-yl)acetyl]amino]ethylamino]-2-oxoethoxy]ethoxy]ethylamino]-3-methyl-1-oxopentan-2-yl]-1-[(2R)-2-[[(2S)-2-aminopropanoyl]amino]-3-sulfanylpropanoyl]pyrrolidine-2-carboxamide

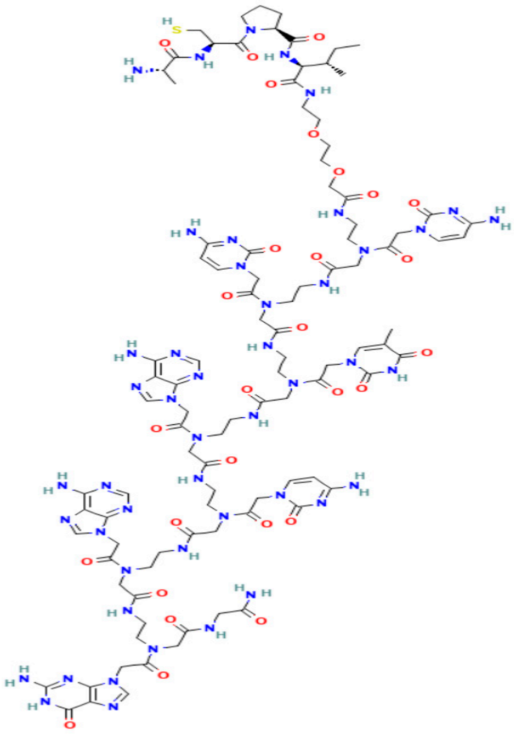

SUPPLEMENTARY FILES

4 (2S)-6-amino-2-[[(2S)-1-[(2S)-2-[[(2S)-2-[[2-[[(2S)-2-[(2-aminoacetyl)amino]-5-(diaminomethylideneamino)pentanoyl]amino]acetyl]amino]-3-carboxypropanoyl]amino]-3-hydroxypropanoyl]pyrrolidine-2-carbonyl]amino]hexanoic acid

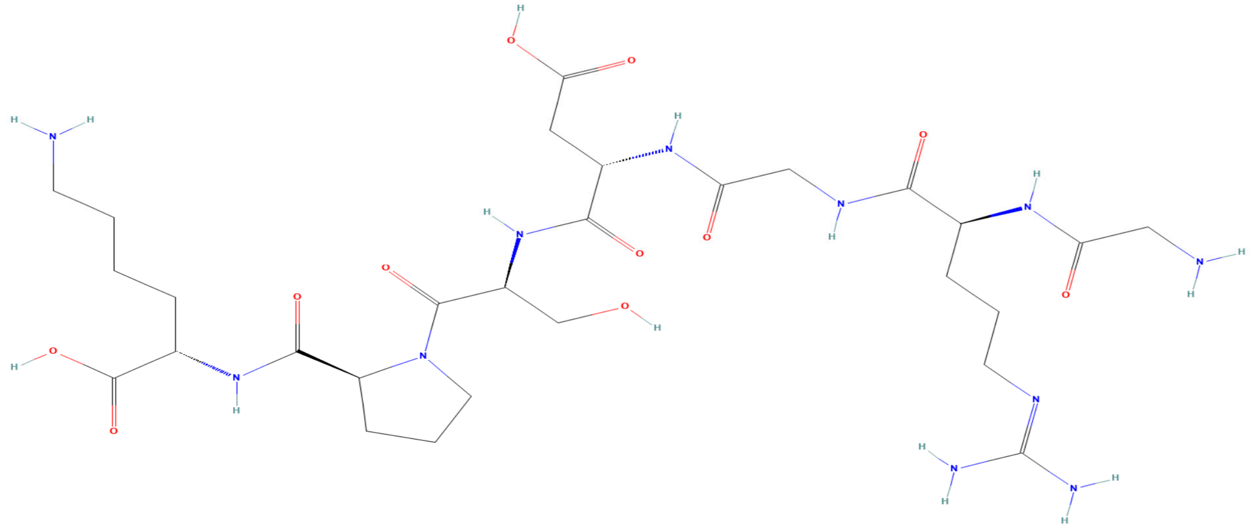

5 (2S)-3-[4-[(2,6-dichlorobenzoyl)amino]phenyl]-2-[[1-[[4-(1-methyltetrazol-5-yl)phenyl]methyl] cyclobutanecarbonyl] amino]propanoic acid

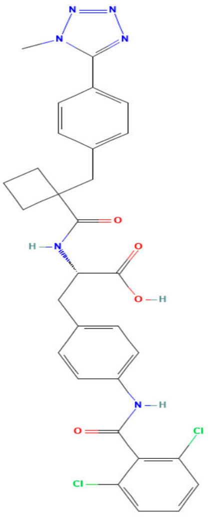

SUPPLEMENTARY FILES

6 (3S)-3-(4-methoxyphenyl)-3-[[2-[3-methoxypropyl-[2-[4-[(2-methylphenyl)carbamoylamino] phenyl] acetyl] amino]acetyl] amino]propanoic acid

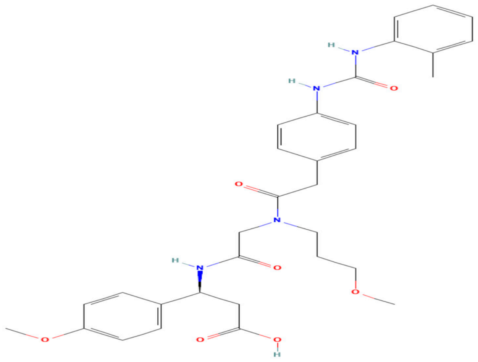

SUPPLEMENTARY FILES

**Table S2:** ADMET parameters for the selected compounds.

| Parameters | Units | Compounds | | | | | |
|---|---|---|---|---|---|---|---|
| | | 1 | 2 | 3 | 4 | 5 | 6 |
| Molecular Weights | Dalton | 2490.0 | 1335.5 | 2465.5 | 715.8 | 607.5 | 590.7 |
| Water solubility | Numeric (log mol/L) | 1.0 | 2.0 | 3.0 | 4.0 | 5.0 | 6.0 |
| Caco2 permeability | Numeric (log Papp in $10^{-6}$ cm/s) | -2.9 | -2.9 | -2.9 | -2.9 | -3.0 | -3.4 |
| Intestinal absorption (human) | Numeric (% Absorbed) | -2.2 | -0.3 | -1.8 | -0.9 | -0.1 | 0.0 |
| Skin Permeability | Numeric (log Kp) | 0.0 | 0.0 | 0.0 | 0.0 | 58.4 | 55.3 |
| P-glycoprotein substrate | Categorical (Yes/No) | -2.7 | -2.7 | -2.7 | -2.7 | -2.7 | -2.7 |
| P-glycoprotein I inhibitor | Categorical (Yes/No) | Yes | Yes | Yes | Yes | No | Yes |
| P-glycoprotein II inhibitor | Categorical (Yes/No) | No | No | No | No | No | No |
| VDss (human) | Numeric (log L/kg) | No | No | No | No | Yes | No |
| Fraction unbound (human) | Numeric (Fu) | 0.0 | -0.3 | 0.0 | -1.6 | -1.6 | -0.3 |
| BBB permeability | Numeric (log BB) | 0.4 | 0.4 | 0.4 | 0.7 | 0.2 | 0.0 |
| CNS permeability | Numeric (log PS) | -6.2 | -4.6 | -7.4 | -2.3 | -1.5 | -1.5 |
| CYP2D6 substrate | Categorical (Yes/No) | -9.9 | -7.8 | -9.5 | -6.6 | -3.0 | -3.4 |
| CYP3A4 substrate | Categorical (Yes/No) | No | No | No | No | No | No |
| CYP1A2 inhibitor | Categorical (Yes/No) | No | No | No | No | Yes | Yes |

SUPPLEMENTARY FILES

| | | | | | | | |
|---|---|---|---|---|---|---|---|
| CYP2C19 inhibitor | Categorical (Yes/No) | No | No | No | No | No | No |
| CYP2C9 inhibitor | Categorical (Yes/No) | No | No | No | No | No | No |
| CYP2D6 inhibitor | Categorical (Yes/No) | No | No | No | No | Yes | Yes |
| CYP3A4 inhibitor | Categorical (Yes/No) | No | No | No | No | No | No |
| Total Clearance | Numeric (log ml/min/kg) | No | No | No | No | No | No |
| Renal OCT2 substrate | Categorical (Yes/No) | 1.1 | 0.8 | 0.2 | 0.3 | -0.2 | 0.3 |
| AMES toxicity | Categorical (Yes/No) | No | No | No | No | No | No |
| Max. tolerated dose (human) | Numeric (log mg/kg/day) | No | No | Yes | No | No | No |
| hERG I inhibitor | Categorical (Yes/No) | 0.4 | 0.4 | 0.4 | 0.5 | 0.5 | 0.6 |
| hERG II inhibitor | Categorical (Yes/No) | No | No | No | No | No | No |
| Oral Rat Acute Toxicity (LD50) | Numeric (mol/kg) | Yes | No | Yes | No | Yes | No |
| Oral Rat Chronic Toxicity (LOAEL) | Numeric (log mg/kg_bw/day) | 2.5 | 2.5 | 2.5 | 2.5 | 2.7 | 2.9 |
| Hepatotoxicity | Categorical (Yes/No) | 11.2 | 3.2 | -1.0 | 3.0 | 2.7 | 1.8 |
| Skin Sensitisation | Categorical (Yes/No) | Yes | Yes | No | No | Yes | Yes |
| *T. pyriformis* toxicity | Numeric (log ug/L) | No | No | No | No | No | No |
| Minnow toxicity | Numeric (log mM) | 0.3 | 0.3 | 0.3 | 0.3 | 0.3 | 0.3 |
